# Supplementary material for: Fluoroalkyl Pentacarbonylmanganese(I) Complexes as Initiators for the Radical (co)Polymerization of Fluoromonomers
Source: Polymers (Basel). 2020 Feb 8;12(2):384. doi: 10.3390/polym12020384 (PMC7077403; doi:10.3390/polym12020384)
Supplement: Supplementary file 1 [file polymers-12-00384-s001.pdf]

polymers

MDPI

Supplementary materials

Fluoroalkyl Pentacarbonylmanganese(I) Complexes as Initiators for the Radical (co)Polymerization of Fluoromonomers

Roberto Morales-Cerrada, Vincent Ladmiral, Florence Gayet, Christophe Fliedel, Rinaldo Poli,* Bruno Améduri*

First-order kinetics plots

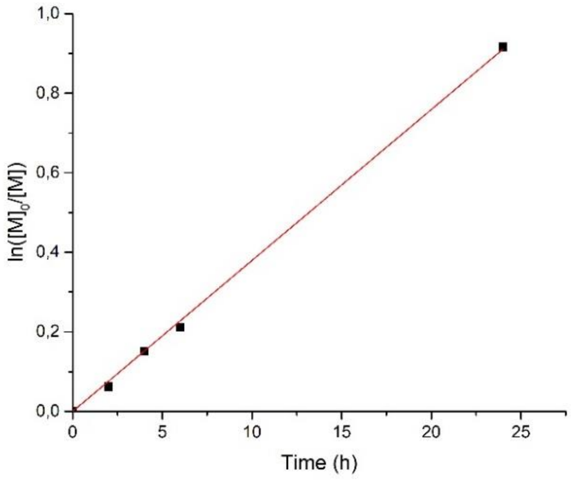

Figure S1. First-order kinetics plot for the polymerization of VDF initiated by visible light in presence of [$Mn(CF_3)(CO)_5$].

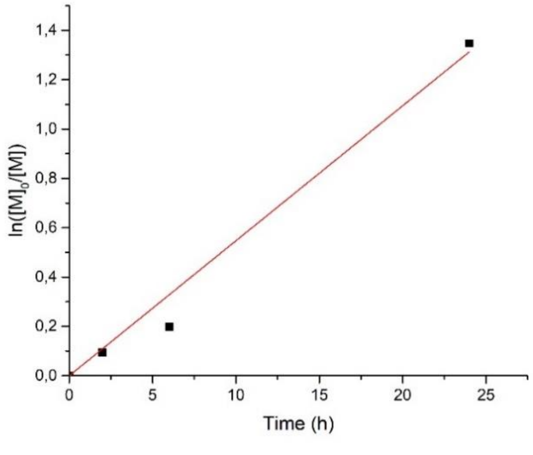

Figure S2. First-order kinetics plot for the polymerization of VDF initiated by UV irradiation (300 nm) in presence of [$Mn(CF_3)(CO)_5$].

SEC chromatograms

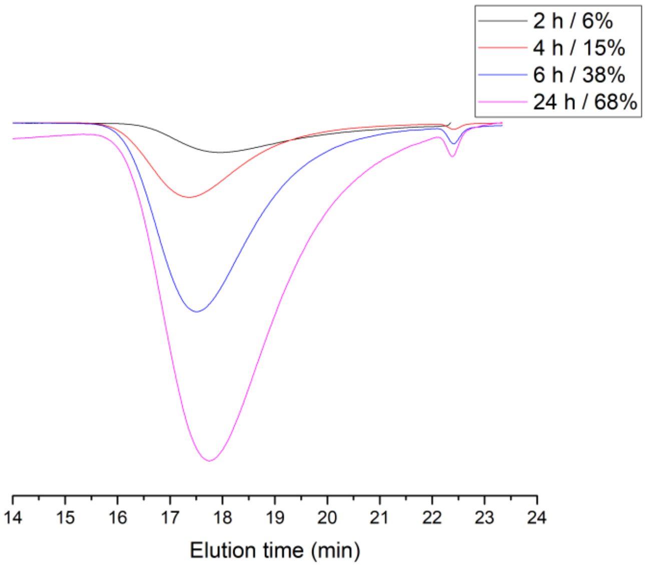

Figure S3. SEC traces of PVDF samples obtained by thermal radical polymerization of VDF with [Mn(CF_3)(CO)$_5$] in DMF normalized with conversion (entries 2, 3, 4 and 7 of Table 1).

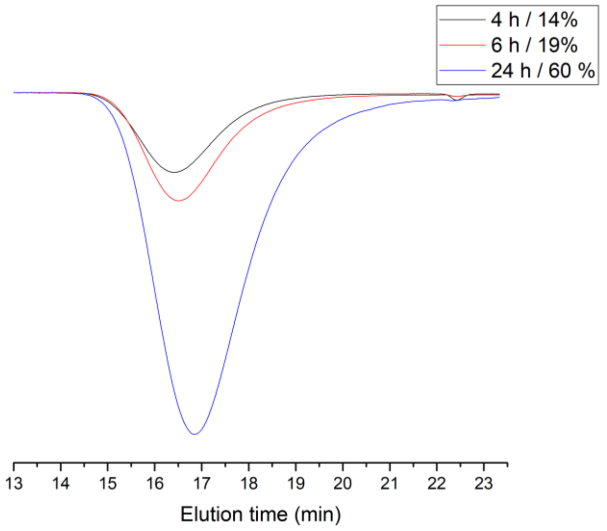

Figure S4. SEC traces of PVDF samples initiated by visible light (entries 9 to 11 of Table 1) in DMF normalized with conversion.

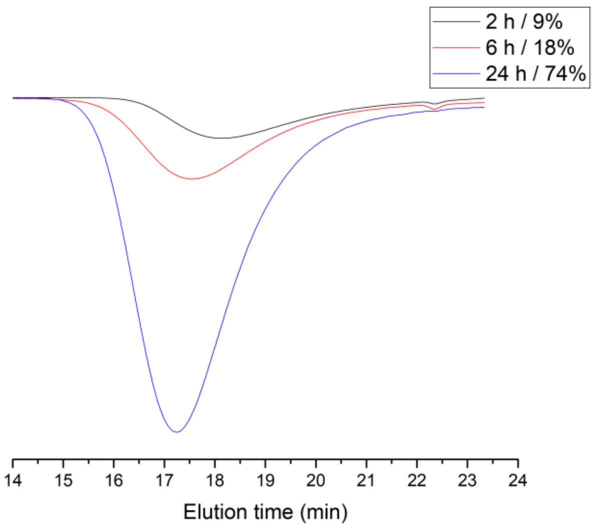

Figure S5. SEC traces of PVDF samples initiated by UV irradiation (entries 12 to 14 of Table 1) in DMF normalized with conversion.

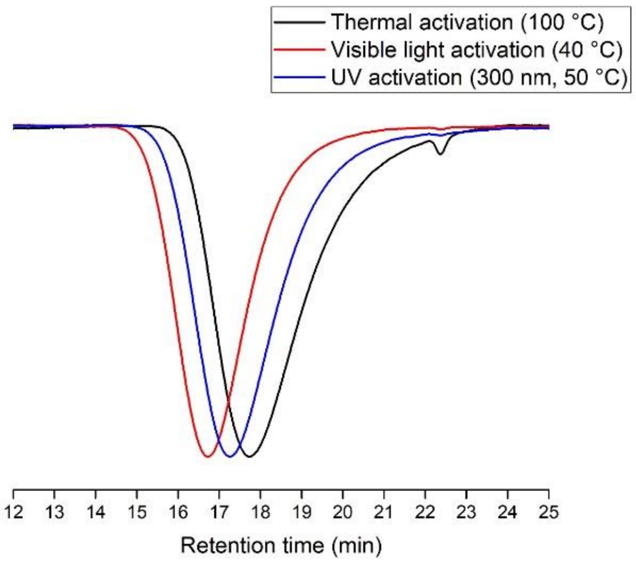

Figure S6. SEC traces of PVDF samples (entries 7, 11 and 14 of Table 1) in DMF after 24 h-reaction by various initiation methods.

Number average molar mass and dispersity vs. conversion plots

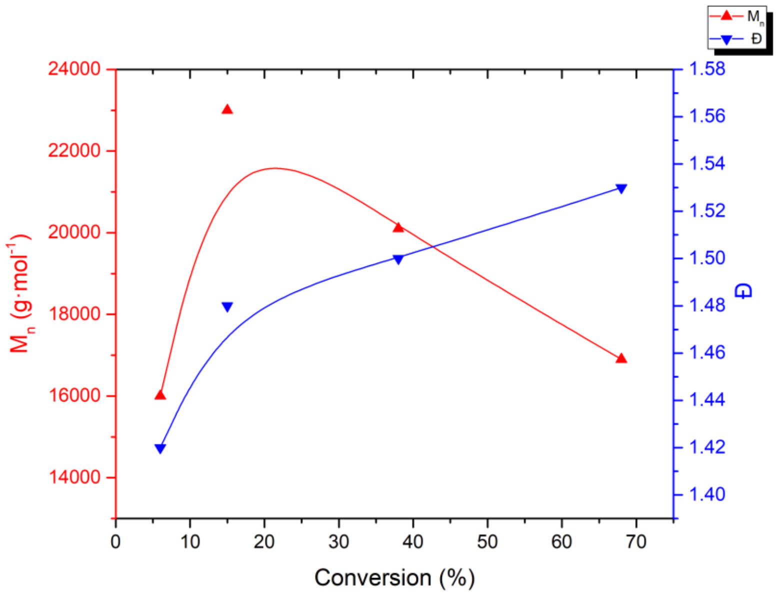

Figure S7. Plot of number average molar mass and dispersity vs conversion of VDF polymerization initiated thermally (100 °C) by complex **1**.

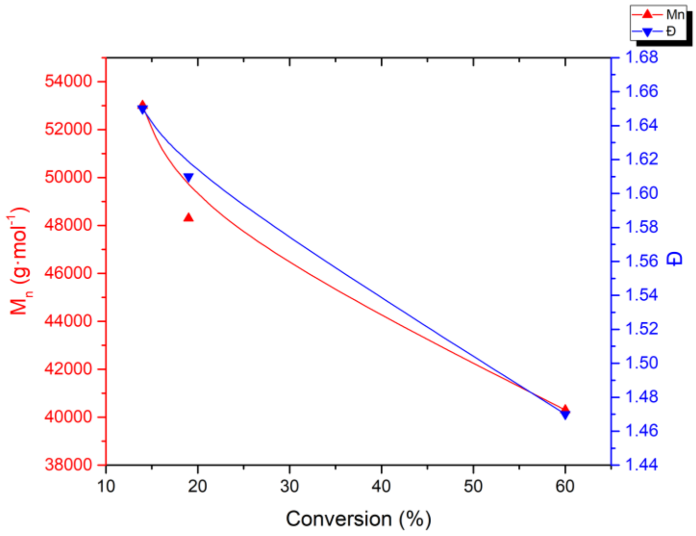

Figure S8. Plot of number average molar mass and dispersity vs conversion of VDF polymerization initiated by complex **1** under visible light irradiation.

NMR characterization

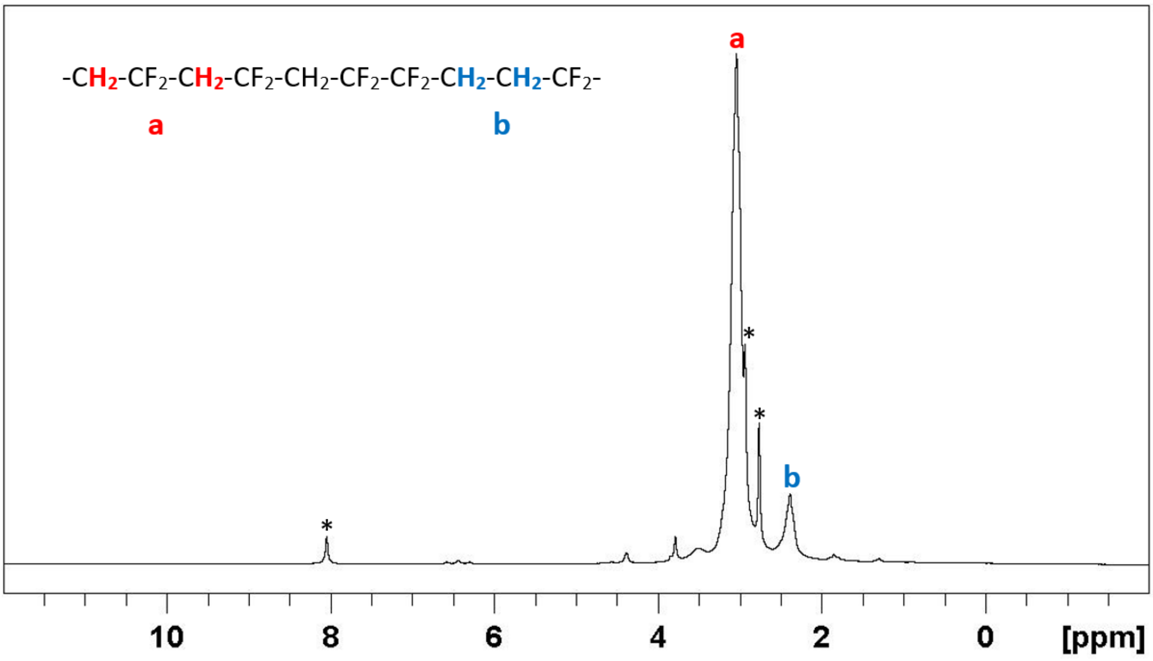

Figure S9. ^{1}H NMR spectrum (400 MHz, DMF-d_7) of the PVDF obtained by thermal activation of **1** (entry 7 of Table 1). The starred resonances are due to the solvent.

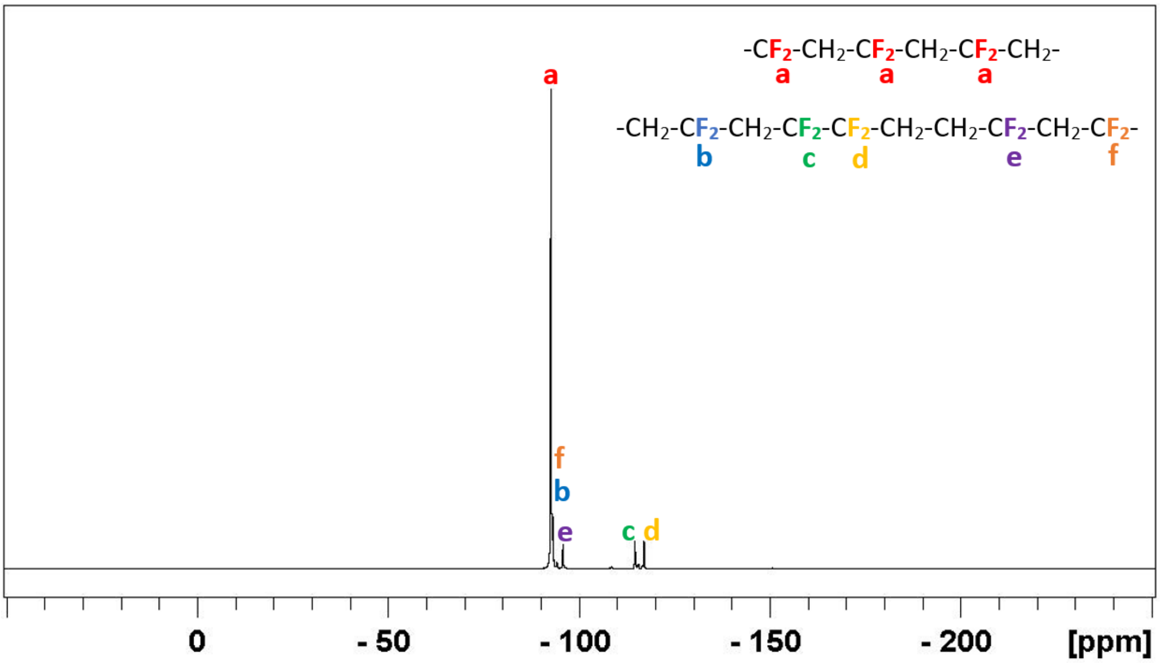

Figure S10. ^{19}F NMR spectrum (376.5 MHz, DMF-d_7) of the PVDF obtained by thermal activation of **1** (entry 5 in Table 1).

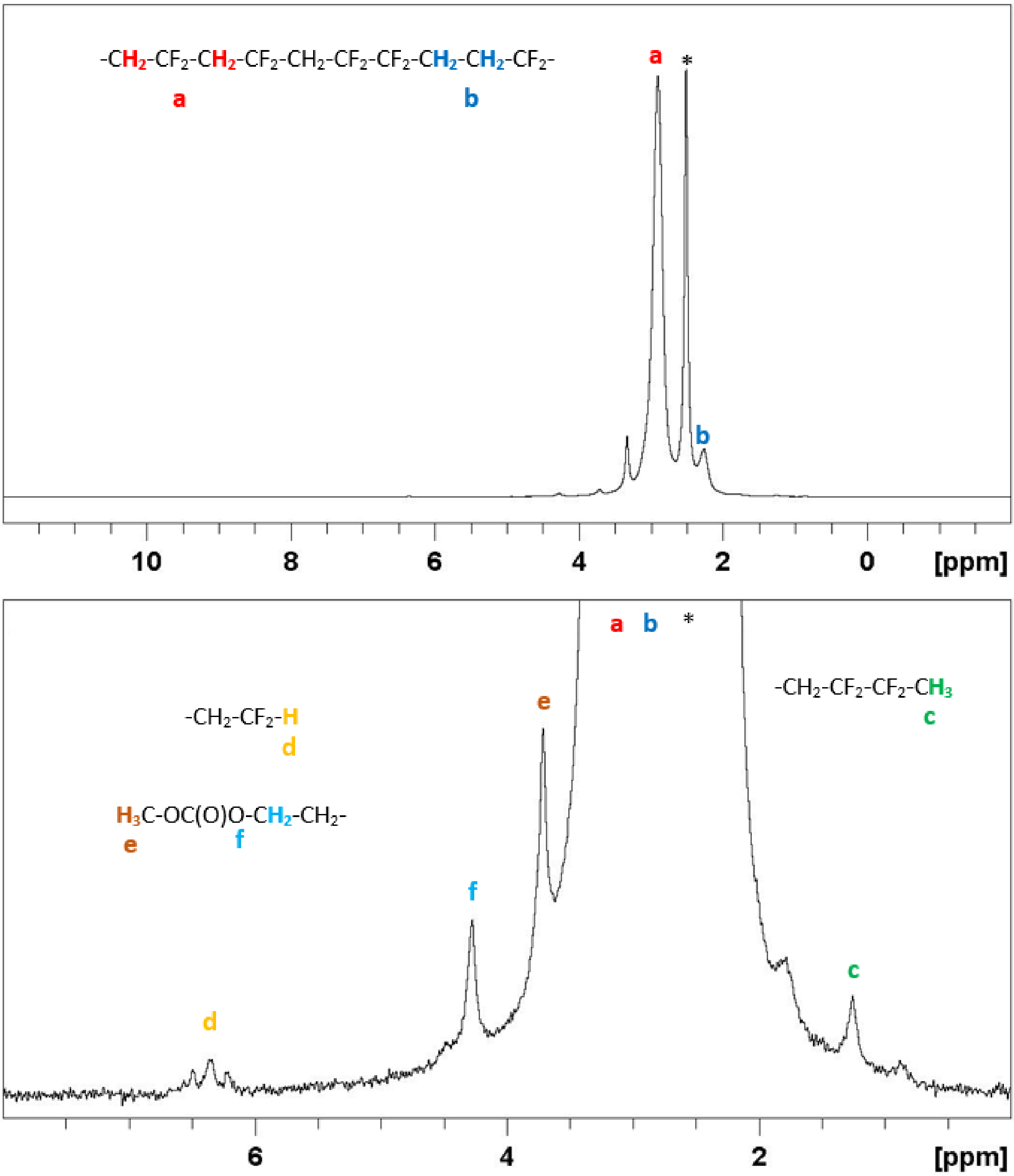

Figure S11. ^{1}H NMR spectrum (400 MHz, DMSO-*d*6) of the PVDF obtained by visible light activation of **1** (entry 11 of Table 1). Full spectrum (above) and expansion of the 0 to 8 ppm region (below). The starred resonances are due to the solvent.

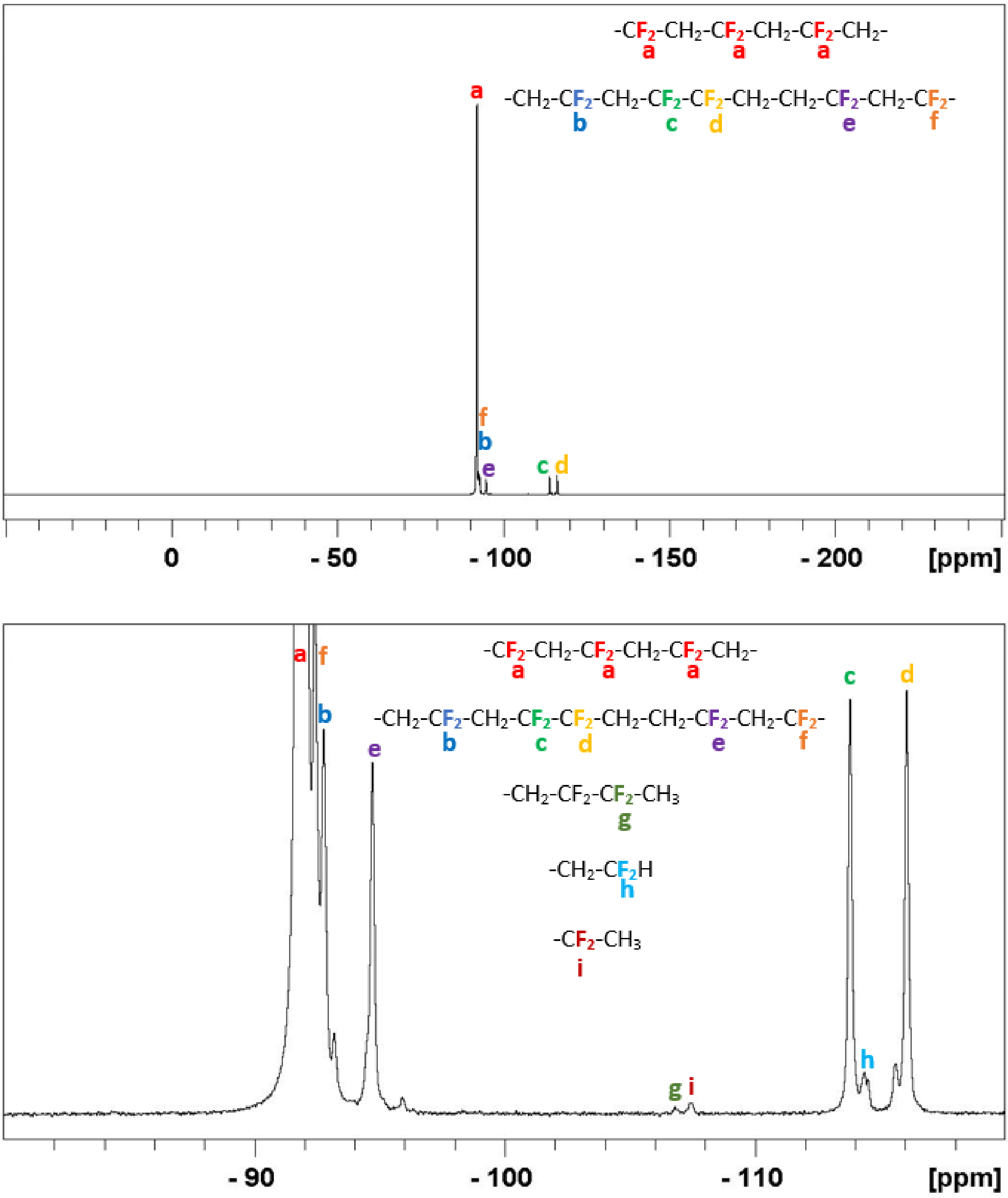

Figure S12. ^{19}F NMR spectrum (376.5 MHz, DMSO-*d*6) of the PVDF obtained by visible light activation of **1** (entry 11 of Table 1). Full spectrum (above) and expansion of the -120 to -80 ppm region (below).

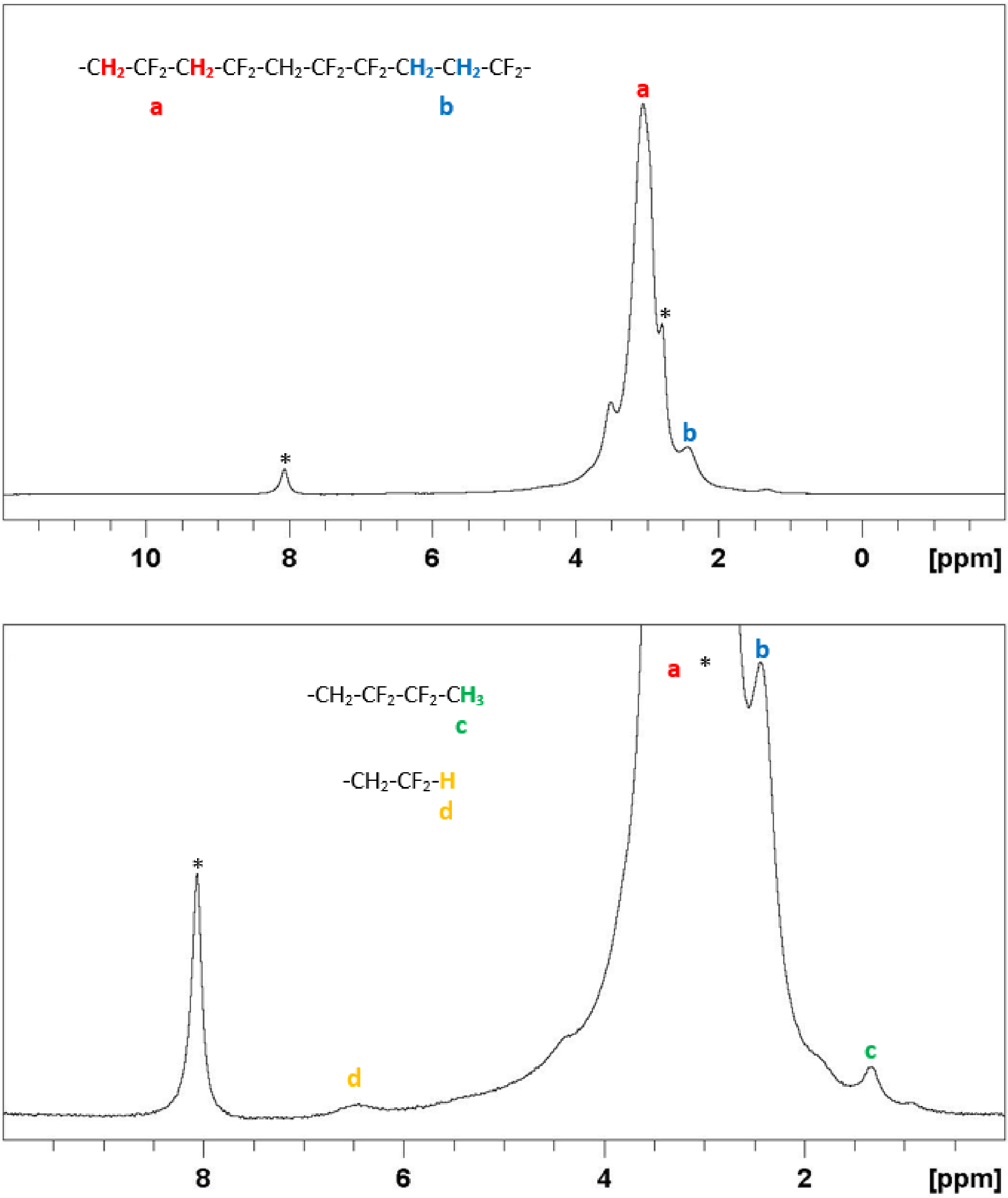

Figure S13. ^{1}H NMR spectrum (400 MHz, DMF-*d*$_7$) of the PVDF obtained by UV light activation of **1** (entry 14 of Table 1). Full spectrum (above) and expansion of the 0 to 10 ppm region (below). The starred resonances are due to the solvent.

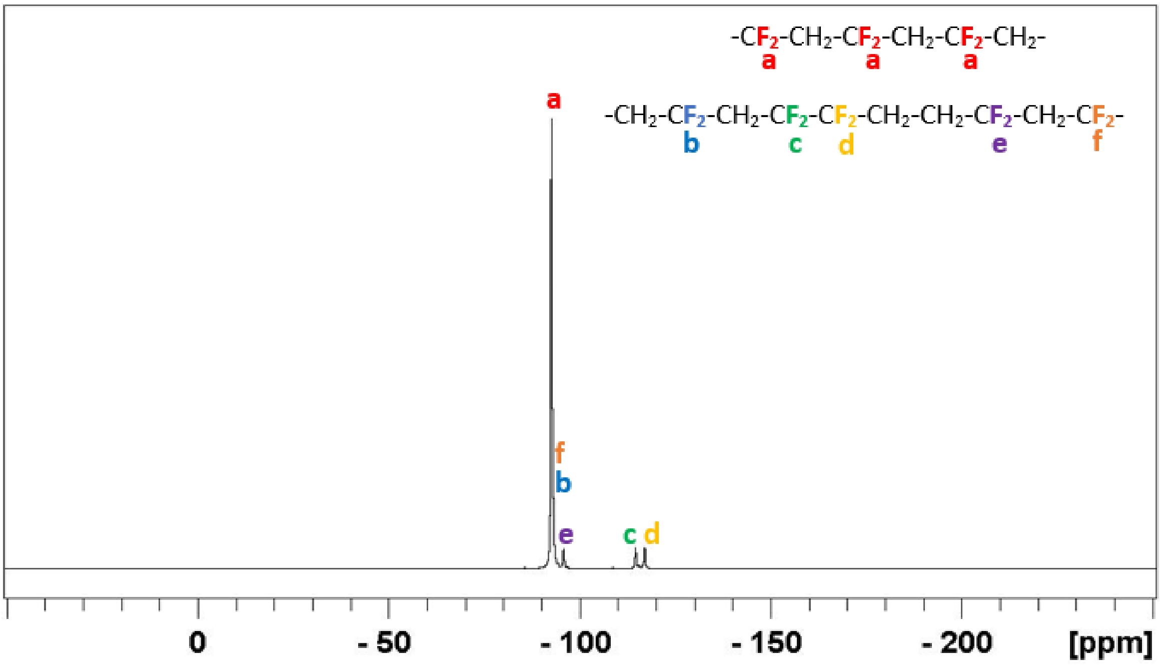

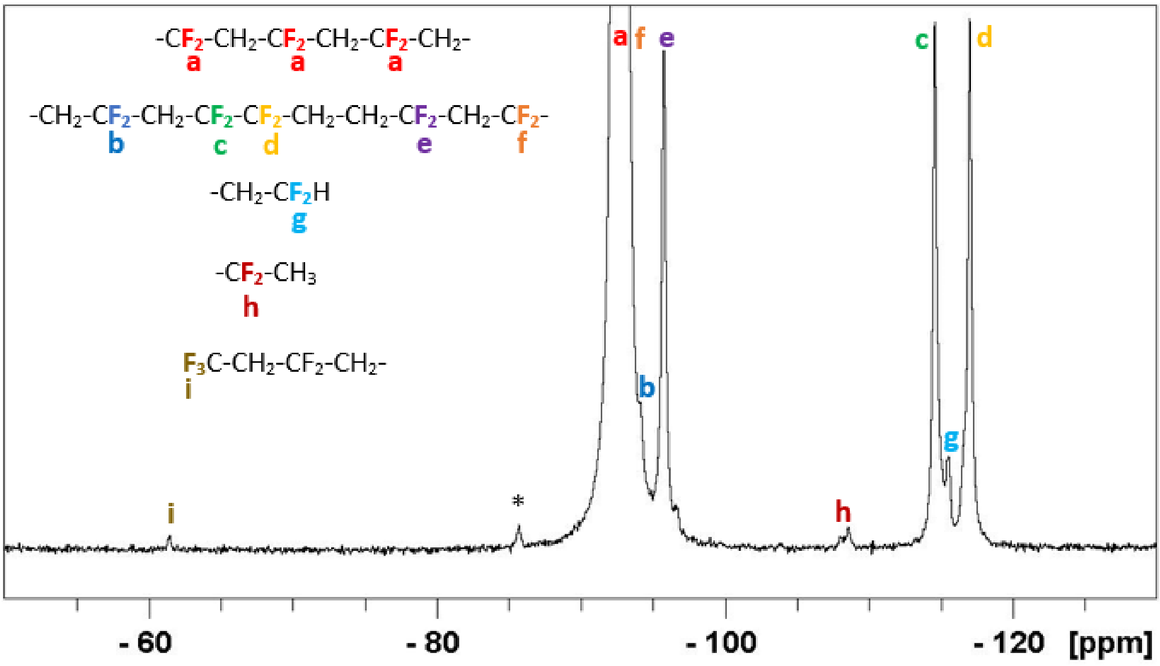

Figure S14. ^{19}F NMR spectrum (376.5 MHz, DMF-d_7) of the PVDF obtained by UV light activation of **1** (entry 14 of Table 1). Full spectrum (above) and expansion of the -120 to -80 ppm region (below). The starred resonance could not be attributed to any expected signal of possible products.

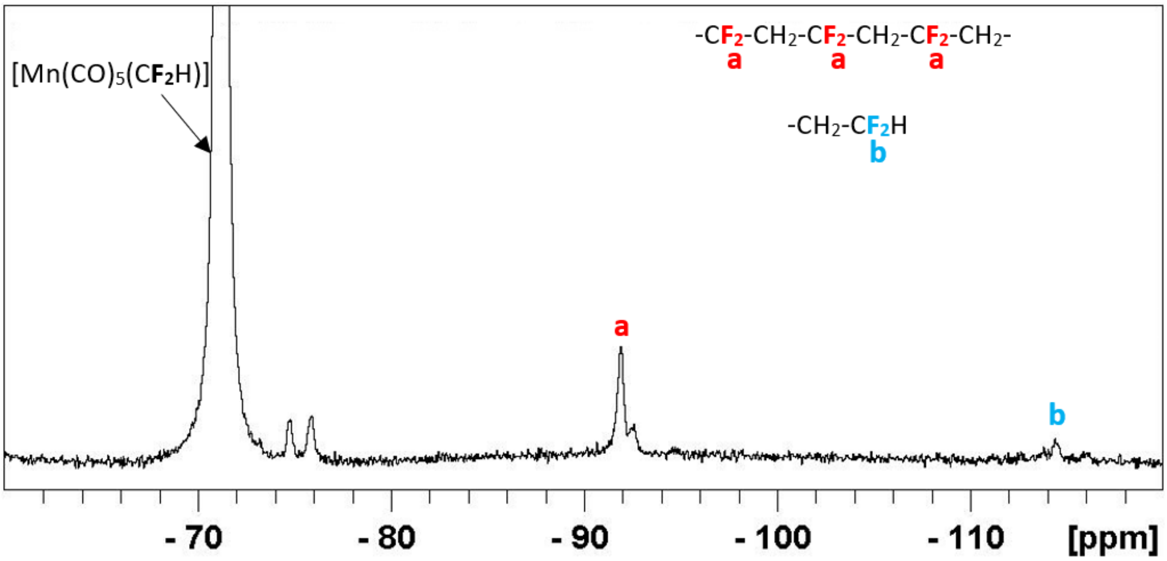

Figure S15. ^{19}F NMR spectrum (376.5 MHz, DMF-*d7*) of the PVDF obtained by visible activation of **2** (entry 2 of Table 4).

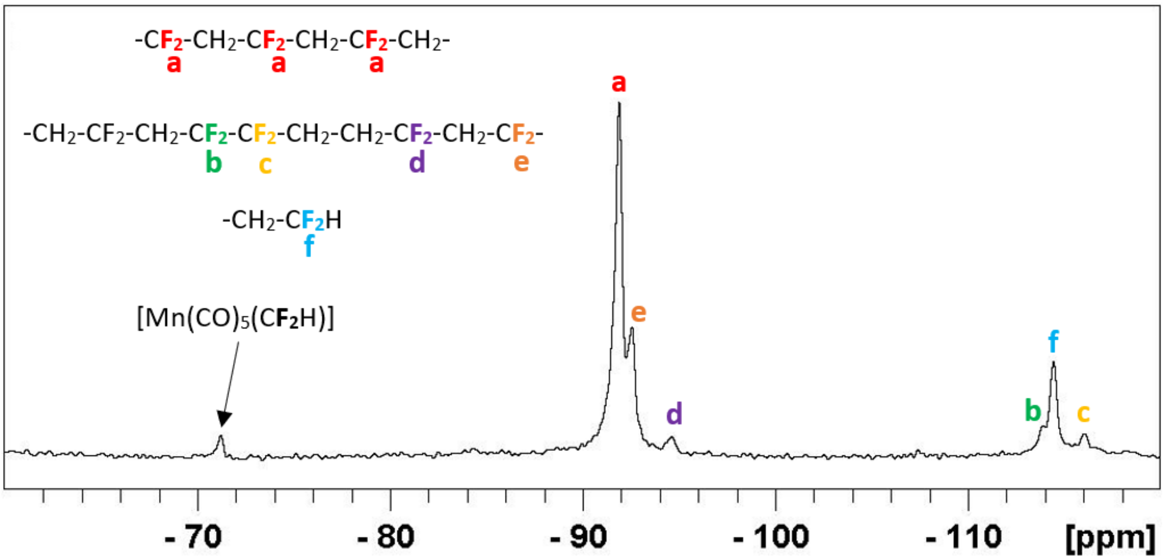

Figure S16. ^{19}F NMR spectrum (376.5 MHz, DMF-*d7*) of the PVDF obtained by UV activation of **2** (entry 3 of Table 4).

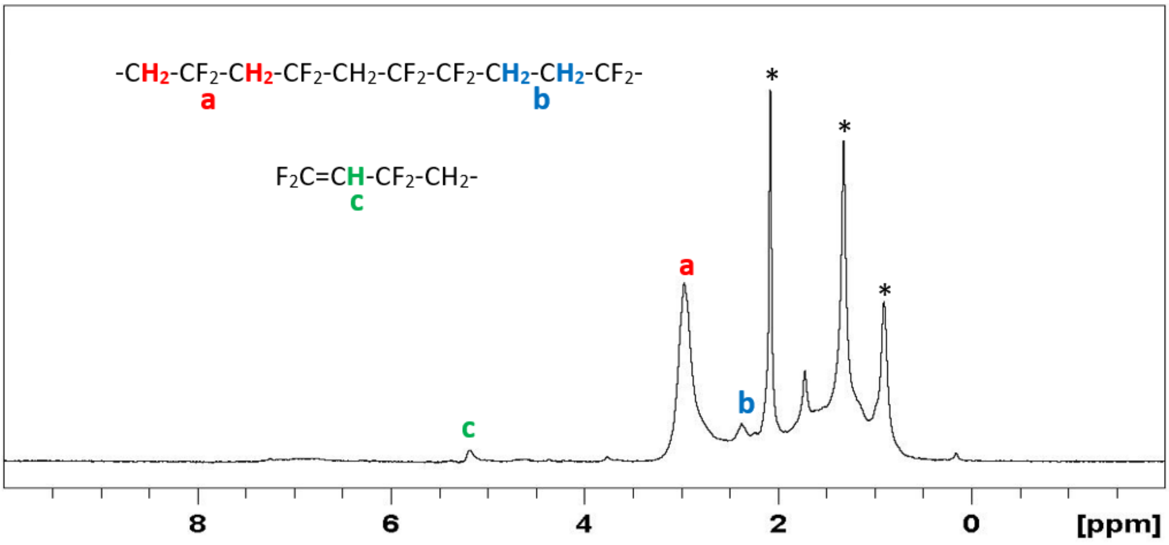

Figure S17. ^{1}H NMR spectrum (400 MHz, acetone-d_6) of the PVDF obtained by thermal activation of **3** (entry 6 of Table 4). The starred resonance is due to the deuterated solvent and precipitation solvent (*n*-pentane).

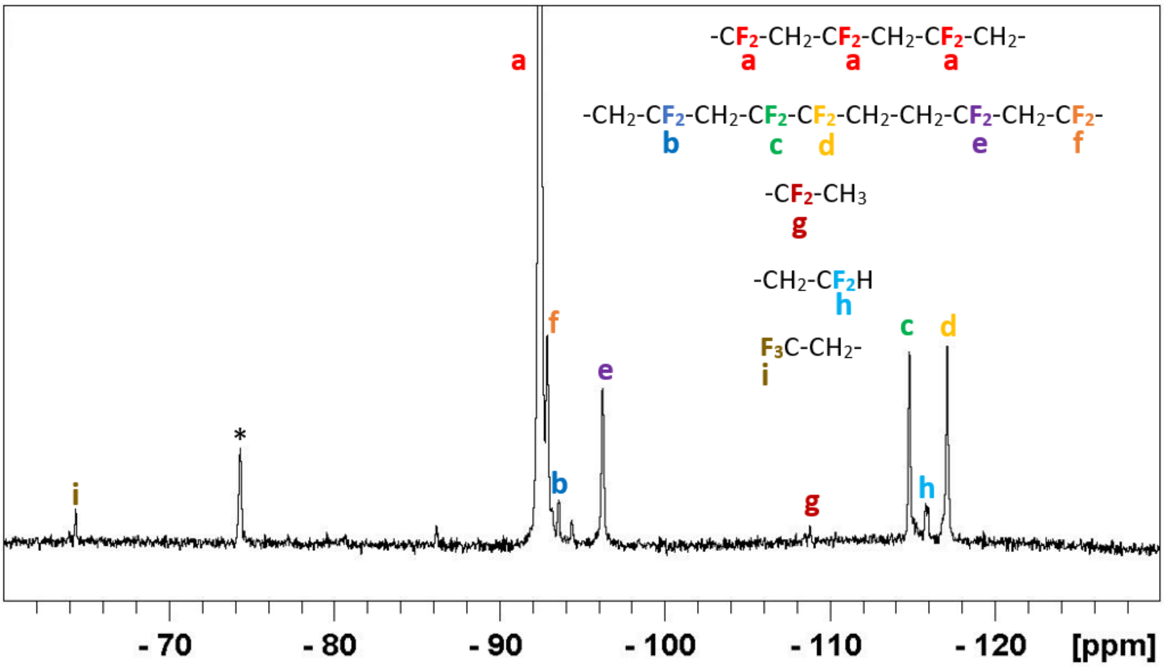

Figure S18. ^{19}F NMR spectrum (376.5 MHz, acetone-d_6) of the PVDF obtained by thermal activation of **3** (entry 6 of Table 4). The starred resonances could not be attributed to any expected signal of possible products.

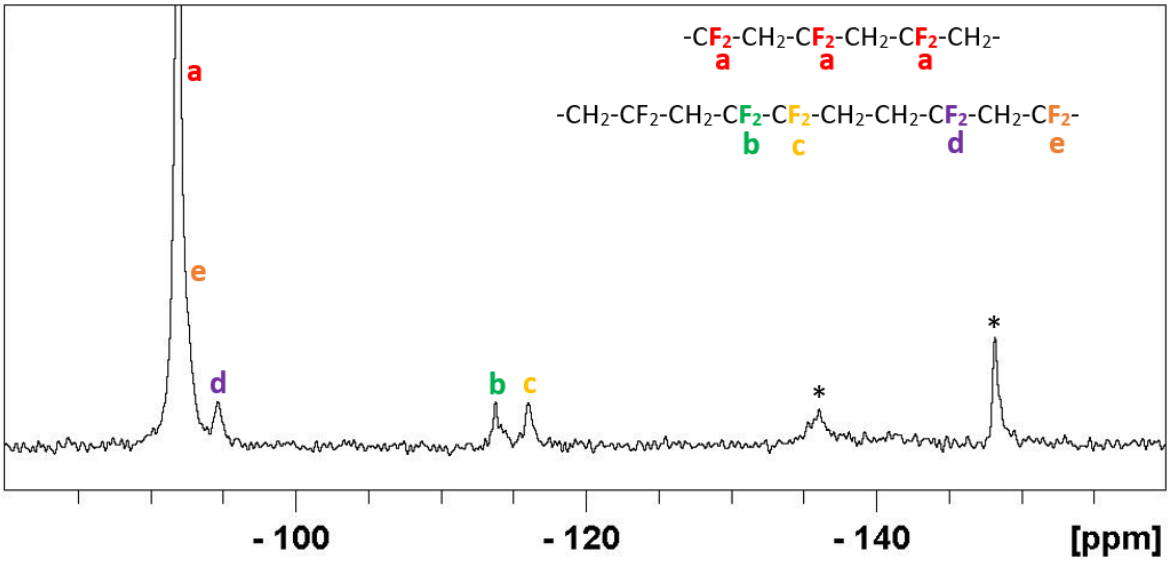

Figure S19. ^{19}F NMR spectrum (376.5 MHz, DMSO-d_6) of the PVDF obtained by thermal activation of **5** (entry 7 of Table 4). The starred resonances could not be attributed to any expected signal of possible products.

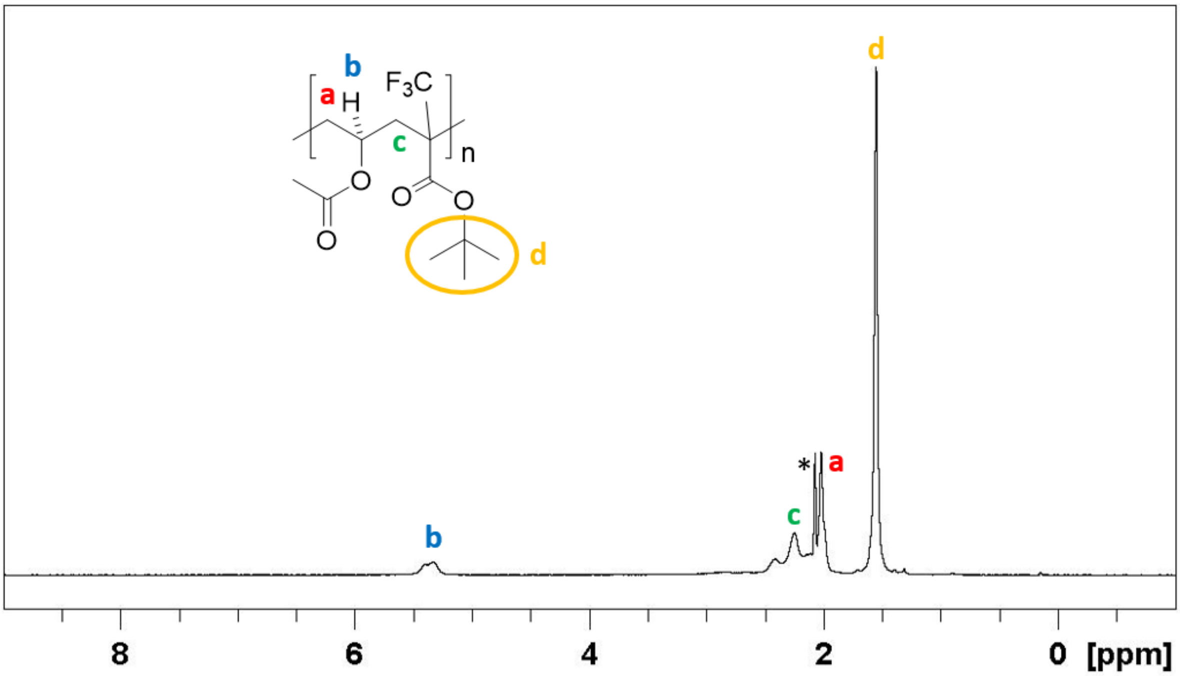

Figure S20. ^{1}H NMR spectrum (400 MHz, acetone-d_6) of the poly(VAc-*alt*-MAF-TBE) obtained by thermal activation of **3** (entry 4 of Table 5). The starred resonance is due to the solvent.

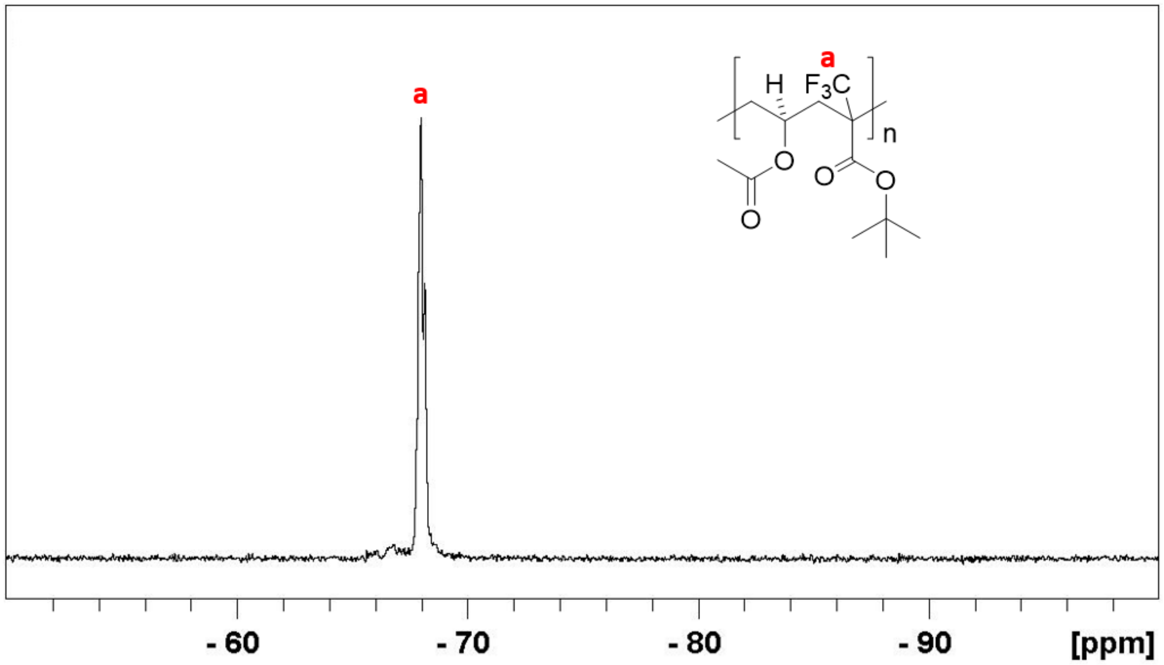

Figure S21. ^{19}F NMR spectrum (376.5 MHz, acetone-d_6) of the poly(VAc-*alt*-MAF-TBE) obtained by thermal activation of **3** (entry 4 of Table 5).

© 2019 by the authors. Submitted for possible open access publication under the terms and conditions of the Creative Commons Attribution (CC BY) license (http://creativecommons.org/licenses/by/4.0/).
